# Nest Population Structure and Wood Litter Consumption by *Microcerotermes indistinctus* (Isoptera) in a Seasonally Dry Tropical Forest, Northeastern Brazil

**DOI:** 10.3390/insects9030097

**Published:** 2018-08-10

**Authors:** Reberth R. B. Barca, Emanuelly F. Lucena, Alexandre Vasconcellos

**Affiliations:** 1 Programa de Pós-Graduação em Ciências Biológicas, Centro de Biociências, Universidade Federal do Rio Grande do Norte, Natal 59072-970, Rio Grande do Norte, Brazil; reberthricelle@yahoo.com.br; 2 Laboratório de Termitologia, Departamento de Sistemática e Ecologia, Universidade Federal da Paraíba, João Pessoa 58051-900, Paraíba, Brazil; emanuellylucenamat@gmail.com

**Keywords:** Caatinga, ecosystem services, nymphoids, Neotropical Region, litterfall

## Abstract

Termites are abundant arthropods in tropical ecosystems and actively participate in the process of litter decomposition. The objective of this study was to evaluate the population structure of *Microcerotermes indistinctus* in arboreal nests and to estimate their contribution to the consumption of wood litter in an area of Caatinga, a type of seasonally dry tropical forest located in the Brazilian semi-arid region. The populations of fifteen nests were quantified and separated into castes, “larvae”, and eggs. Wood blocks of four typical Caatinga species were offered to termites under laboratory conditions. Litter production was estimated in the area over the course of one year. The mean population size of *M. indistinctus* was 73,897 individuals/nest, while the mean nest density in the area was 25 active nests/ha. Total consumption of the four types of wood was estimated to be 10.5 mg of wood/g termite (fresh weight)/day. Based on consumption and population size, *M. indistinctus* consumes 0.35% of the total litter and 1.71% of the annual production of branches and twigs in the area. Wood-consuming termites are highly affected by anthropogenic disturbances in the semi-arid region of Brazil, putting the ecosystem services they perform related to the process of litter decomposition at risk.

## 1. Introduction

Termites are eusocial insects with a predominantly tropical and subtropical distribution [1]. Although they are known primarily for the damage they cause to the urban environment and agriculture, the great majority of termite species play an important role in the decomposition of organic matter and in the formation and composition of soils in tropical ecosystems [2,3]. Termites consume a wide variety of organic materials, such as wood, grasses, herbs, litter fungi, lichens, leaves, roots, animal excrement, and even decaying animal carcasses [4]. However, a considerable number of termite species consume wood at some stage of decomposition, thereby exerting a strong influence on the cycling of nutrients present in this resource [4].

Estimates of population sizes and rates of resource consumption by termites can provide essential information regarding the role of these insects in the dynamics of nutrient cycling and energy flow in ecosystems [5]. The construction of conspicuous arboreal and epigeal nests eventually facilitates population estimates in ecosystems, but the vast majority of species—about 65% of the total termite richness of South American humid forests—do not construct such nests [6]. Thus, there are major difficulties in estimating population sizes for an entire termite assemblage. Even for the conspicuous nest-building species, some of the individuals do not stay continuously inside the nest and forage on the most varied substrates [7,8]. This likely explains why there is little quantitative data for termite populations, especially of Neotropical ecosystems [7,8,9,10,11,12,13].

The objectives of this study were to evaluate the population structure of *Microcerotermes indistinctus* Mathews, 1977 (Termitidae, Termitinae) in conspicuous arboreal nests, and to estimate their contribution to the consumption of wood litter in an area of Caatinga, a type of Seasonally Dry Tropical forest located in the semi-arid region of Brazil. In the Neotropical Region, there are quantitative data for populations of *M. indistinctus* (treated as *M. exiguus*) [8], *M. septemtrionalis* Light, 1933 [9], and *M. strunckii* (Sörensen, 1884) [8,12]. *M. indistinctus* occurs in Brazil and Trinidad and Tobago [14]. The species was initially mistakenly identified as *M. exiguus* from the Atlantic Forest and Caatinga [6,8]. In the Caatinga, *M. indistinctus* is widely distributed and can have more than 20 active nests/ha [15,16].

## 2. Materials and Methods

### 2.1. Study Area

The study was developed at Fazenda Cauaçu, a private area of approximately 700 ha (05°34′00.8″ S, 035°55′03.1″ W), located in the municipality of João Câmara, Northeast Brazil. The area comprises a continuum of vegetation composed of secondary forests that lose practically all their leaves during the dry season, have structures ranging from shrubby to arboreal, and possess different histories of anthropic disturbance and levels of recovery. The dominant plant species in the area are *Croton sonderianus* Muell. Arg. (51.1%), *Cenostigma pyramidale* (Tul.) Gagnon & G.P. Lewis (13.0%), *Aspidosperma pyrifolium* Mart. & Zucc. (9.5%), *Mimosa tenuiflora* (Willd.) Poir. (6.0%), and *Mimosa caesalpiniifolia* Benth. (3.5%). The climate of the region is semiarid with an average annual rainfall of 648.6 mm and a short rainy season from March to June. The average annual temperature is 24.7 °C, with a minimum temperature of 21 °C and a maximum of 32 °C, while the mean relative humidity of the air is 70% [17]. The area is located in Ecorregião da Depressão Sertaneja Setentrional (Northern Sertaneja Depression Ecoregion) [18].

### 2.2. Nest Density

The nest density for *Microcerotermes indistinctus* was estimated from fifteen 100 × 20 m transects randomly distributed over an area of 3 ha and separated by at least 50 m. All conspicuous and active nests were counted and the species responsible for the construction of each identified. Nest volume was estimated by water displacement in a vessel or by the formula for a hemi-ellipsoid. Nests that were built surrounding a tree trunk/branches of the trunk were also estimated and subtracted from the nest volume [13].

### 2.3. Colony Size

Fifteen nests of *M. indistinctus* were selected randomly in an area of 3 ha. The nests were always collected between 11:00 and 13:00 h and packed in 50-liter plastic bags. In the laboratory, the nests were measured and weighed. The collected individuals were fixed in FAA solution (37% formalin:acetic acid:ethanol = 1:1:3) for 24 h and subsequently transferred to 80% ethanol for storage. Ten subsamples of 5 g each were extracted from each nest for population estimates, including total number of individuals, biomass, castes present (soldiers, workers and reproductives), larvae, and eggs. Statistical analyses were only performed for nests that had eggs, from the queen’s posture or from replacement reproductives.

### 2.4. Quantification of Wood Consumption in the Laboratory

Quantification of wood consumption by *M. indistinctus* was performed in 25 nontoxic plastic containers with 1.5 liters of capacity. The substrate was composed of 1 cm of expanded vermiculite, 2 cm of sterilized sand, and 60 mL of distilled water [5]. The sand was previously maintained in an oven at 150 °C for 24 h. The vermiculite was inserted into the substrate to maintain moisture inside the containers.

The plant species offered to termites under laboratory conditions were: *C. sonderianus*, *C. pyramidale*, *M. tenuiflora*, and *A. pyrifolium*. These species were chosen because they are common in the Caatinga and because they exist in the study area [19,20,21]. All the wood had been in the litter for at least two years.

For all the plant species, 1 × 1 × 2.5 cm wooden blocks were made. Two longitudinal holes in the direction of the wood fibers were made with the aid of an electric drill and a 1.5 mm diameter steel drill bit. All of the blocks were placed in an oven and heated at a constant temperature of 105 °C for 72 h. The blocks were then weighed and identified with a small aluminum plate (1 cm^2^) attached to the blocks with copper wires.

To avoid the eventual proliferation of fungi, the blocks of wood were kept from direct contact with the substrate of the container by placement on 4.5 × 4.5 cm bases made out of aluminum. Four blocks of each type of wood were affixed perpendicular to the base, thereby forming 25 “kits” with four types of wood.

Subsamples (1 g) of termites were carefully inserted into each container. The subsamples were extracted from nests collected in the same study area and transported to the laboratory in a styrofoam box. The subsamples all contained the ratio of 1 soldier:20 workers, which is close to the ratio found in mature colonies (see results). Of the 25 containers, five were chosen randomly as the control group (without termites) and the other 20 contained termites. All 25 containers were kept for 20 d in total darkness with an average ambient temperature of near 26 °C. At the end of the experiment, containers that had greater than 50% mortality were disregarded in the quantification of wood consumption, as was adopted by Vasconcellos and Moura [5]. Under laboratory conditions, the decrease in the number of individuals (subsamples) and the maintenance of a constant temperature represent the main changes in relation to the natural environment.

The wood consumption rate was calculated as the difference between the initial and final weight of the blocks, with the value corrected if necessary by the weight lost by the control wood. The consumption obtained in each container was transformed into mg of dry wood consumed/g termite fresh weight/day. The comparison of wood consumption was performed using a one-way ANOVA with a posteriori Tukey test.

### 2.5. Litter Production

Litterfall was collected each month from November 2010 to October 2011 in a 1 × 1 m collector net composed of a galvanized steel frame suspending a nylon mesh (1.0 mm) approximately 20 cm above the ground at the center of each plot. The nylon mesh enabled the falling litter to be collected without the accumulation of water (and thus avoided decomposition during the rainy season). The litter produced was collected monthly and separated into leaves, reproductive structures, twigs, branches, and miscellaneous. The material was placed in an oven at 70 °C for 4 days and then weighed on a precision scale.

## 3. Results

### 3.1. Nest Population

An average of 25.3 ± 14.8 (±SD) active nests/ha was recorded for *M. indistinctus*, representing 53.9% of the total number of conspicuous nests in the area. In addition to *M. indistinctus*, four other species of termites also had conspicuous nests: *Constrictotermes cyphergaster* (Silvestri, 1901) (16.7 ± 12.8 active nests/ha), *M. strunckii* (2.7 ± 3.7), *Nasutitermes corniger* (Motschulsky, 1855) (0.7 ± 1.8), and *N. macrocephalus* (Silvestri, 1903) (1.7 ± 3.1). The mean density for all species was 47.0 ± 20.3 active nests/ha.

The mean population size for *M. indistinctus* nests was 73,897 ± 51,529 individuals, with a range of 6758 to 160,107/nest (Table 1). The mean biomass per nest was 78.6 ± 51.5 g (fresh weight), with a range of 7.1 to 172.8 g (fresh weight)/nest. The estimated mean density of *M. indistinctus* was 187 individuals/m^2^ and 0.34 g termite (fresh weight)/m^2^, corresponding to 1,869,770 individuals/ha and a biomass of 3.4 kg (fresh weight)/ha, respectively.

**Figure 1:**
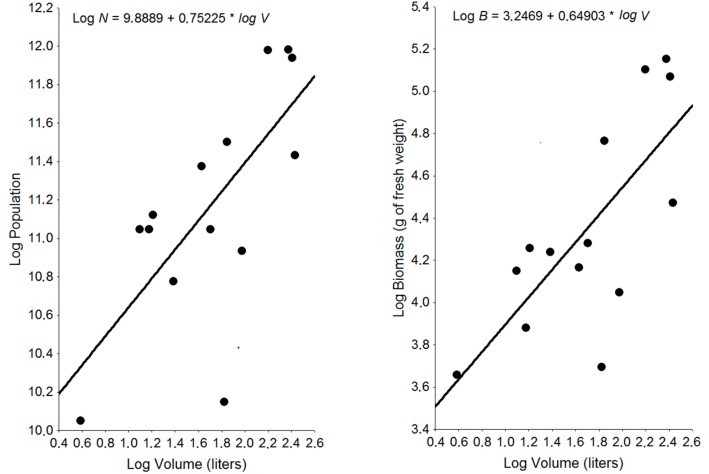
Relationship between population parameters and volume of 14 nests of *Microcerotermes indistinctus*, at Fazenda Cauaçu, João Câmara, Rio Grande do Norte, Brazil.

The queen was found in nine nests, but the presence of eggs was recorded in 14 nests. Only nest B was egg-free and clearly in decline. In one of the nests without a queen, nest *G*, the presence of 14 replacement reproducers was recorded, all nymphoids. Winged individuals were found in only four nests. The number and biomass of individuals were related to nest volume (*r_number_* = 0.70; *n* = 14; *p* < 0.01) and (*r_biomass_* = 0.73; *n* = 14; *p* < 0.01), respectively (Figure 1). Nest weight and volume were significantly related (*r* = 0.95; *n* = 14; *p* < 0.01). In numerical terms, the young forms (“larvae” 1, “larvae” 2, white soldiers and nymphs) ranged from 7.9 to 47.8% of all the individuals, with a mean of 37.7% of the total per nest. The number of soldiers per nest ranged from 1.2 to 4.3% of all the individuals, with a mean of 2.5% (Table 1). Nest B exhibited some different parameters in relation to the others, such as a detachment of the relationship between the number of individuals and the volume of the nest. The ratio of soldiers to workers (1:78) in this nest was also quite different from that of the other nests.

### 3.2. Quantification of Wood Consumption in the Laboratory

The total consumption of the four types of wood by *M. indistinctus* was estimated at 10.5 ± 3.6 mg of wood/g of termite (fresh weight)/day. There was a significant difference in the consumption of the different types of wood (F_1,3_ = 25.4; *p* < 0.001), with the consumption of *C. pyramidale*, of 5.2 ± 1.7 mg of wood/g of termite fresh weight/day, being higher than that for all the other tested plant species (Table 2).

### 3.3. Wood Litter Production and the Impact of M. indistinctus

The total litter production was 3673 mg/ha/year. There was a predominance of leaf production, which represented approximately 72% of total dry weight. Branches and twigs represented approximately 20%, while reproductive structures and miscellaneous materials represented approximately 6% and 2%, respectively. Based on the consumption rate and population size for *M. indistinctus* in the area, the average consumption rate was 35.9 g/ha/day or 13.1 kg/ha/year. This value represents a consumption of 0.35% of the total litter produced and 1.71% of the annual production of branches and twigs in the area.

## 4. Discussion

The mean population size of nests of *M. indistinctus* was within the range recorded for other species of the genus in the Neotropical Region, as well as in the Afrotropical Region [8,12,22]. As with *M. insdistinctus*, populations of *M. strunckii* nests in the Neotropics can reach over 140,000 individuals [12]. The population size estimated for the nests of *M. indistinctus* may be even greater, since some individuals were certainly out of the nest, foraging among the soil, leaves, and dead wood, when sampling took place. In the Atlantic Forest of Northeast Brazil, only 13.7% of individuals of *Anoplotermes banksi* Emerson, 1925 were inside their nests, with the remainder (86.3%) being in the soil profile or in tree trunks in advanced states of decomposition (46.1%) [8].

In Neotropical ecosystems, the nest density for *M. indistinctus* can exceed 20 nests/ha and reach more than 50 nests/ha in the Atlantic Forest. In this same ecosystem, there are no more than three nests/ha of *M. strunckii* [6,8,23]. In the Afrotropical Region, the nest density of *M. edentatus* Wasmann, 1911 can exceed 100 nests/ha, while in the Oriental Region, the maximum nest density for *M. biroi* (Desneux, 1905) was approximately 50 nests/ha (review in Lepage and Darlington [22]).

The relationship between nest volume and population size was only moderately strong for *M. indistinctus*. The absence of a strong (>90%) relationship between these variables can be explained by three factors: (i) At the moment of sampling, a significant portion of the population is outside the nest actively foraging; (ii) the nest population is declining, senescent; and (iii) when the quantified nest is polycalic, that is, the population may be unevenly distributed between the interconnected subunits of the nest [7,24,25,26].

Fourteen nymphoid-type reproductives were found in nest G, representing the first recorded case for *M. indistinctus*. There are records of neotenics for at least 18 species of *Microcerotermes* Silvestri 1901, principally nymphoids and ergatoids, found in the field or obtained in the laboratory after orphanage experiments (review in Myles [27]). In the Neotropical Region, there have only been records of neotenic forms for *M. arboreus* Emerson, 1925 and *M. strunckii*. In Argentina, colonies of *M. strunckii* with nymphoids and/or ergatoids of both sexes were found, but only the nymphoids were physogastric [12]. The population of nest B exhibited several characteristics of senility, such as low percentages of “larvae”, as well as a marked decline in the rate of egg laying by the queen, resulting in a progressive population decline [22].

The consumption values recorded for *M. indistinctus* (10.5 mg of wood/g of termite/day) in the present study were relatively high when compared to the consumption rates recorded for other species of the family Termitidae under laboratory conditions, which varied from 2.0 to 12.2 mg of wood/g of termite/day [4], and low when compared to a species of a temperate region, *Reticulitermes flavipes* (Kollar, 1837), with 50 mg of wood/g of termite/day [28]. Vasconcellos and Bandeira [29] recorded the consumption of 6.62 mg of wood/g of termite/day for a relatively common species of *Microcerotermes* in an area of Atlantic Forest in Northeast Brazil. In the Caatinga, the highest consumption of vegetal biomass was recorded for *Heterotermes sulcatus* Mathews, 1977, with 78 mg/g of termite/day [30], which is seven times higher than that found for *M. indistinctus*. In this same ecosystem, there are also records of the consumption of vegetal biomass by *Constrictotermes cyphergaster*, obtained from the difference in weight of the workers before and after the foraging period, which revealed an estimated consumption of 13 mg/g of termite/day (recalculated value) [31].

The plant species most consumed by *M. indistinctus* was *C. pyramidale*, an abundant species widely distributed in the Caatinga, and especially in the Depressão Sertaneja Setentrional ecoregion [19,20,21]. Several properties of wood offered under laboratory conditions have been found to influence the rate of consumption by termites. Such properties include the chemical composition (amount of carbohydrates, nitrogen and secondary compounds) and physical attributes (hardness and density), which may facilitate chewing and assimilation of the nutrients present in the substrate [4,32]. Given that the wood blocks were oven dried at 105 °C for 72 h, some natural properties of the wood were lost and this possibly represents an influence factor in the consumption rate of the termites. However, we believe that this can have an effect by reducing the consumption rate rather than increasing it.

The participation of *M. indistinctus* in the consumption of vegetal necromass produced may seem relatively low, but in other Caatinga areas within the same ecoregion, there may be up to 15 species (39 to 57% of all the species) of wood consuming termites [15,33], including *H. sulcatus* and *C. cyphergaster*, substantially increasing the participation of termites in the removal of the annual production of vegetal necromass. In savanna ecosystems, termites can consume 5% to more than 80% of the primary production, with intense participation of species of Macrotermitinae [3,34]. In the Atlantic Forest of Northeast Brazil, only *N. corniger*, *N. ephratae*, and *N. macrocephalus*, of a total of 18 spp. of wood-feeders, removed between 2.9 and 3.3% of the total annual production of necromass of all branches and twigs. The consumption rate was based on the block of wood, and this may represent a source of bias in extrapolations for branches and twigs. Estimates for entire communities of termites are quite difficult, but Collins estimated the participation of termites in plant necromass consumption to range from 14.7 to 16.3% of all resources produced in an area of humid tropical forest in Malaysia [35]. In temperate forests, the participation of termites, especially *Reticulitermes* spp., represents 10% of annual wood necromass inputs [28].

Municipalities in the Caatinga possess low values for the Human Development Index (HDI) and the human population still maintains a strong dependence on natural resources extracted directly from ecosystems [36]. Anthropogenic disturbances caused by the removal of plant biomass and soil compaction from trampling by goats and cattle significantly affect the termite assemblage of the Caatinga, with more pronounced effects on wood feeders and constructors of conspicuous arboreal nests [15], as is the case for *M. indistinctus* and *C. cyphergaster*. This fact may alter the participation of these organisms in the process of wood decomposition, thus compromising the cycling of the nutrients present in this substrate.

## Figures and Tables

**Table 1:** Composition of 15 nests of *Microcertotermes indistinctus* analyzed in an area of Caatinga at Fazenda Cauaçu, municipality of João Câmara, Rio Grande do Norte, Northeast Brazil. Descriptions of biomass and the ratios given in the table are exclusive to soldiers and workers. Rat.: ratio; Rep.: reproductive; Pop.: population; We: weight; V: volume; Ind.: number of individuals; B: biomass; L: “larvae”; W: winged; S: secondary reproductives (neotenics); Q: queen; M: mean; SD: standard deviation.

			Soldier		Worker		Rat.				Rep.		Pop.
Nest	We (kg)	V (L)	Ind.	B (g)	Ind.	B (g)	S/W	L ^1^	E ^2^	W	S	Q	Total
A	1.2	3.4	209	0.5	3643	6.6	1:17	2906	2857	-	-	1	6758
B	4.3	9.5	116	0.3	9008	16.2	1:78	116	-	-	-	1	9240
C	2.6	6.2	1962	4.3	19,953	35.9	1:10	3615	188	-	-	1	25,530
D	1.7	4.0	1364	3.0	36,907	66.4	1:27	9493	523	93	-	-	47,857
E	4.0	11.1	5992	13.2	81,010	145.8	1:13	55,407	10,895	77	-	1	153,381
F	0.7	1.8	1013	2.2	20,329	36.6	1:20	1,838	646	-	-	-	23,180
G	3.4	10.8	4077	9.0	91,004	163.8	1:22	65,012	36,646	-	14	-	160,107
H	3.9	11.4	2471	5.4	45,706	82.3	1:18	44,154	13,913	-	-	-	92,331
I	2.0	5.5	1593	3.5	38,281	68.9	1:24	22,898	20,210	-	-	1	62,772
J	1.9	7.2	947	2.1	30,647	55.2	1:32	24,488	8431	-	-	-	56,082
L	1.1	3.3	1021	2.3	25,618	46.1	1:25	36,113	18,918	95	-	1	62,847
M	1.1	3.0	1076	2.4	33,881	61.0	1:31	27,851	3050	35	-	1	62,843
N	1.7	5.1	1330	2.9	34,236	61.6	1:25	51,665	1330	-	-	-	87,231
O	3.4	9.0	5044	11.1	85,344	153.6	1:16	69,088	20,740	-	-	1	159,476
P	2.5	6.4	1982	4.4	62,728	112.9	1:31	34,125	24,213	-	-	1	98,835
M	2.4	6.5	2013	4.4	41,220	74.2	26	29,918	11,611				73,897
SD	1.2	3.2	1720	3.8	27,144	48.9	16	23,521	11,237				51,529

^1^ Although “larvae” were counted in the population, their biomass was not calculated; ^2^ eggs were not counted in the total population.

**Table 2:** Consumption of wood in mg/g termite fresh weight/day by *Microcerotermes indistinctus* under laboratory conditions.

Plant Species	Density (g/cm^3^)	Range of Wood Consumption	Mean Wood Consumption ^1^
*Cenostigma pyramidale*	0.96	2.7–76	5.2 ± 1.7 ^a^
*Mimosa tenuiflora*	0.92	1.1–3.8	2.4 ± 0.8 ^b^
*Croton sonderianus*	0.72	0.9–3.0	1.5 ± 0.6 ^b^
*Aspidosperma pyrifolium*	0.77	0.8–2.6	1.4 ± 0.5 ^b^

^1^ Different letters indicate statistically significant differences according to the Tukey test.
